# Effective image fusion strategies in scientific signal processing disciplines: Application to cancer and carcinoma treatment planning

**DOI:** 10.1371/journal.pone.0301441

**Published:** 2024-07-12

**Authors:** Ayush Dogra, Bhawna Goyal, Dawa Chyophel Lepcha, Ahmed Alkhayyat, Devendra Singh, Durga Prasad Bavirisetti, Vinay Kukreja

**Affiliations:** 1 Chitkara University Institute of Engineering and Technology, Chitkara University, Punjab, India; 2 Department of ECE and UCRD, Chandigarh University, Mohali, Punjab, India; 3 College of Technical Engineering, The Islamic University, Najaf, Iraq; 4 Department of Computer science & Engineering, Uttaranchal Institute of Technology, Uttaranchal University, Dehradun, India; 5 Department of Computer Science, Norwegian University of Science and Technology, Trondheim, Norway; University of the Punjab, PAKISTAN

## Abstract

Multimodal medical image fusion is a perennially prominent research topic that can obtain informative medical images and aid radiologists in diagnosing and treating disease more effectively. However, the recent state-of-the-art methods extract and fuse features by subjectively defining constraints, which easily distort the exclusive information of source images. To overcome these problems and get a better fusion method, this study proposes a 2D data fusion method that uses salient structure extraction (SSE) and a swift algorithm via normalized convolution to fuse different types of medical images. First, salient structure extraction (SSE) is used to attenuate the effect of noise and irrelevant data in the source images by preserving the significant structures. The salient structure extraction is performed to ensure that the pixels with a higher gradient magnitude impact the choices of their neighbors and further provide a way to restore the sharply altered pixels to their neighbors. In addition, a Swift algorithm is used to overcome the excessive pixel values and modify the contrast of the source images. Furthermore, the method proposes an efficient method for performing edge-preserving filtering using normalized convolution. In the end,the fused image are obtained through linear combination of the processed image and the input images based on the properties of the filters. A quantitative function composed of structural loss and region mutual data loss is designed to produce restrictions for preserving data at feature level and the structural level. Extensive experiments on CT-MRI images demonstrate that the proposed algorithm exhibits superior performance when compared to some of the state-of-the-art methods in terms of providing detailed information, edge contour, and overall contrasts.

## 1 Introduction

Medical professionals (radiologists) need a higher level of spatial and spectral detail in the images for numerous applications such as monitoring, research, accurate disease analysis, and the therapy. The computed tomography (CT) images are often utilised to show bone information but there is a lack of information regarding magnetic resonance imaging (MRI) images and soft tissues, which allows for the acquisition of this type of data using a single modality image [1, 2]. In addition, positron emission tomography (PET) images provide details of the soft tissues along with scare boundary details. To address this issue, supporting evidence from additional modalities is needed. In this context, the term “fusion” represents to a technique for combining multiple multimodalities images. A technique known as image fusion combines important details from images to produce a single fusion image that comprises more detailed info than one or more information than the source photographs [3, 4]. The few applications that utilise this fused image include multi-focus image fusion, robot vision, satellite imaging, aerial imaging, and medical imaging [5, 6]. Image fusion is the practice of utilizing specialized techniques to combine multiple different images to produce a novel image [7, 8]. The data extraction from images received from several sources is what it entails [9, 10]. The creative multi-spectral images have a higher spatial resolution while still retaining their spectral details [11, 12]. The three stages of image fusion are decision, feature, and the pixel level fusion. The large parts of the significant data are retained in a composite image in case of pixel level fusion. When combining different characteristics at the feature-level, the sources of those features such as edges and textures are mainly considered [13]. The act of incorporating a final decision is known as decision-level fusion. Two different types of image fusion exist. The fusion of single sensor and the multisensory photographs brings together an individual image from the number of sensors to produce a fused image and their individual photographs are combined to produce a fusion image. One such technique is the merger of several information and details [14, 15].

Multi-sensor image fusion is the procedure of fusing several photographs from numerous sensors to produce a composite image by considering the military or medical imaging [16]. In addition to biometrics, machine vision, military applications, medical imaging, automatic change detection, navigational aids, remote and aerial sensing, satellite imaging and digital imaging, image fusion is used in the wide range of other fields as well. Medical image fusion is also applicable in the domain of clinical circumstances such as diagnosis and therapy. Medical image fusion considered to combines multiple images together to provide a single detailed image. The computer vison is used in medical image fusion approaches [17, 18]. The PET, MRI, SPET, magnetic resonance angiography (MRA), ultrasonography (USG), multimodality medical imaging includes procedures like CT and MRI etc. Higher resolution images are produced by structural based therapeutic imaging techniques, namely CT, USG, MRA and MRI. The functional therapeutic imaging techniques like functional MRI (*f*MRI) and SPECT generate low-resolution photographs with functional details. The functional and anatomical therapeutic photographs may be combined to develop a highly insightful understanding of a given object. Medical image fusion lowers storage costs by saving a single fusion image instead of a group of source images [19]. Different imaging methods only provide some information. An image from a CT scan could show the precise bone formations. Both healthy and unhealthy soft tissues can be found with a soft tissue MRI scan. It is probable to reduce replication and increase diagnosis precision by combining complimentary data from CT and MRI images [20, 21]. Clinical diagnosis and treatment can be carried out using structural data and PET/MRI imaging [22, 23]. In multimodal medical image fusion, fusion at the pixel level is used [24].

Several medical image fusion techniques [24, 25] has been developed within last few decades. A robust method to fuse multiple images related to a pixel-based fusion rule was proposed [26, 27]. This method employs a hybridization of cross bilateral filter (CBF) and edge-aware filtering. This method produces clear and clean fused images free of noise and artefacts while maintaining key details from multiple images. Hu et al. [28] propose an algorithm related to separable dictionary learning and the Gabor filter, and Polinati et al. [29] present a fusion algorithm based on local energy maxima with empirical wavelet decomposition. Li et al. [30] present a robust method based on segment graph filtering with sparse representation. Recently, a fusion method with reduced computational complexity for improving target detection accuracy and providing the foundation for clinical diagnosis was proposed [31]. Lepcha et al. [32] introduce a significantly improved fusion of medical images related to anisotropic diffusion (AD) and CBF using pixel significance. Kaur et al. [33] introduces a fusion of medical images based on a multi-objective differential evolution-related deep neural network. When compared to other existing methods, both methods generate excellent results by strongly combining multiple images and ensuring robustness in several quantitative parameters. But the fusion outcomes of all these algorithms are still prone to blurring, noise, and distortions. A fusion technique based on RBFNN and DSWT was proposed and evaluated by Chao et al. [34]. First, they use 2 level decomposition to divide each photograph into 7 parts, each of which includes both low-and high frequency components to analyse the specifics or feature details of two images that will be processed by DSWT. They replaced the remaining sections in the similar position in the two photographs utilising the proposed upgraded RBFNN while considering the gradient and energy features of the target.

Yousif et al. [35] present a smart fusion method based on sparse representation and convolutional neural network (CNN). First, entire input images are fed into the standard OMP where the sparse representation (SR) fused photograph is produced utilizing max fusion rule which tries to enhance pixel localisation. Second, for every source image, a novel k-singular value decomposition (K-SVD) dictionary learning strategy based on SCNN is used once more. The approach has demonstrated good non-linear behaviour, which has helped to improve the fused output’s sparsity characteristics and show improved image detail extraction and transfer. The MSDRA network is a novel convolutional neural network that Li et al. [36] suggest being used to fuse anatomical and functional medical images. The network comprises of an image restoration mode, feature extraction mode and the feature fusion mode. In the feature extraction mode, we extract image characteristics by sequentially connecting three identical MSDRA blocks. There are two branches in the MSDRA block. However, a second branch utilises 3×3 convolutional kernels, the first branch employs a multiscale technique for extracting features at various scales using three convolutional kernels of various sizes. To combine the characteristics collected from the input photographs, authors propose the feature L1 norm fusion technique. The HMMIF method is proposed for pathologic research on conditions like neurocysticercosis, degenerative illnesses, and cancer [37]. In this research, two domain models based on HMMIF methods are proposed to use in MRI-CT, PET-MRI, and SPECT- MRI image fusion applications. The proposed strategy first divides source images into high and low frequency coefficient utilising nonsubsampled contourlet transform (NSCT). For NSCT coefficients with low frequency, the mean fusion rule is applicable. The process of complete fusion causes the high-frequency components of NSCT decomposition to fuse. The directed filtration method is utilised to address the high frequency of NSCTs. A novel image synthesis technique put forth by P.H. Dinh enables effective process even when an input photograph is noisy or contains weak contrast [38]. First, it introduces a novel enhancement technique that concentrates on resolving the noise of source image or low contrast issues. The CLAHE, BM3D, and chirp scaling algorithm (CSA) are the few techniques used to build this enhancement algorithm. Based on the adaptive parameters gleaned from the suggested image improvement approach, it then introduces a technique to divide an image into three enhanced layers. The input photograph is splits into high-and low frequency layers using this image decomposition technique.

An innovative method to combine multimodal medical images has been proposed by Goyal et al. [39] using anisotropic diffusion (AD) and NSCT. To coarsely separate two aspects for source images, namely a textural and structural details. The approach first uses AD to decomposes input photographs into their detail and the base layers. A sum based fusion process is used to further integrate the detail and base layers. This rule successfully preserves most of the structural and textural details while maximizing the noise filtering contrast level. These photographs are subsequently splits into low-and high frequency parts using NSCT. Further, by independently supporting eigenfeature reinforcement in the fusion results, these coefficients are integrated using a principal component analysis/ Karhunen-Loeve (PCA/KL) based fusion rule. A multiresolution analysis based on NSCT is carried out fused salient feature details and the contrast enhanced fusion components. The ultimate fusion result is created by applying an inverse NSCT to each coefficient. An image fusion technique is presented by Phu-Hung Dinh [40]. The supplied photographs first go through pre-process to improve their performance, The three-layer image decomposition (TLID) method is next introduced which splits an image into three separate layers: the base layer *L*_*B*_, a small-scale structure layer *L*_*SS*_, and the large-scale structure layer *L*_*LS*_. To ensure that resultant image is not degraded we then synthesize the basic layers using adaptive principles based on marine predators’ algorithm (MPA). Finally, the paper suggests a productive synthesis technique for *L*_*SS*_ and *L*_*LS*_ layers based on fusing local energy function with its variations. The finer information included in the original photograph are retained by this fusion method.

Zhang et al. introduces unsupervised learning integration network to solve the issues with the prior fusion approach such as a pair of feature difference guided network (FDGNet) [41]. The modified multivariable I-function (MMIF) is modelled as feature weighted guided learning in which feature extraction model is committed for computing the difference between features at numerous levels such that feature restoration model could produce the fusion result. To appropriately train the proposed network a hybrid loss that combines feature difference loss and weighted fidelity loss is also added. The dimension fusion edge-guided network (DFENet) is an image fusion system which combines CNN feature learning with vision transformer feature learning employing self-supervised learning [42]. It is built on the encoder decoder network that could be train on sizable dataset of natural photographs without the requirement for meticulously compiled original fusion images. The novel global semantic details integration module is introduced for effectively combines multiscale characteristics generated by the transformer module that improves performance of the restored images and avoids usage of simple up sampling and concatenate process. The 6 convolutional layers along with two skip connections are composed in decoder, which is used to reconstruct from fused features. The multiscale dense residual attention network (MDRANet) is introduced by Fu et al. and used for the merging of MRI and nuclear medicine images [43]. To extract and improve deep features, MDRANet integrates a MDNet with a MRANet Moreover, MDRANet is optimized the fusion performance is enhanced by utilising four different loss functions. The image decomposition technique based on on quasi-cross bilateral filtering (QBF) is introduced by Zhang et al. [44]. An image is divided into two layers: an energy layer with only information about intensity and the structural layer with detailed information. Thus, to increase the benefits of edge counter extraction, increase sharpness of edge counter and completely preserve photograph detailed information, the visual saliency detection map (VSDM) is employed to direct the fusion of energy levels. The structure layer was fused using IMLMG and the WSEML, where it significantly increased counter edge sharpness. A unique end to end unsupervised network for fusing of medical images was recently presented by Liu et al. [45]. It is made up of two symmetrical discriminators and a generator. The former focuses on producing a “real-like” fused image related to the decisively determined information and structural loss whilst the latter are committed for identifying the discrepancies between the fused photograph and the original ones. They receive alternative training up until a point where discriminators are unable to explain the fusion image from the source ones. A further benefit of the symmetrical discriminator approach is that it helps to preserve feature consistency across various modalities. More notably, U-Net is selected as the generator heuristically for enhancing retention level of texture information and the up-sampling technique is updated to bilinear interpolation to prevent checkerboard artifacts.

In this study, a medical image fusion strategy for improving the quality of numerous types of medical images using a swift algorithm and normalized convolution was proposed. A salient structure extraction (SSE) is used to extract salient structures by reducing the influence of noise and irrelevant information. The structure detection process is utilized for approximating the gradient magnitude using absolute values, whereas the mean filter is utilized to ensuring that the pixel with greater gradient intensity impacts in its neighbours and provides a way to distribute the sharply changed pixels to their neighbours. We further present an efficient method for performing image edge-preserving filtering using normalized convolution. The method maintains geodesic distances across curve points by adaptively warping an input signal, allowing an edge-preserving filter to be carried out in linear time. The fusion outputs are generated by the linear combination of the edge preserving filter results and the input images. The mathematical complexity of our method is lower compared to other competing techniques.

In summary, the main contributions of the proposed method are as follows:

1. This study proposes a method to combine two-dimensional data using salient structure extraction (SSE) and a swift algorithm through normalized convolution. This method gets rid of noise and extraneous details by combining the most important parts of images from different types of medical imaging images.
2. The method implements high-quality edge-aware image filtering using a dimensionality reduction method. It leads to filters with different desirable properties and notably speeds up the fusion operation.
3. In addition, the output of normalised convolution is combined with the source images using a weighted-sum rule to produce the fusion result, which rearranged image pixels into their original dynamic range for restoring large-scale structures.
4. The proposed method can achieve high-quality image restoration and further boost generalization for different types of imaging modalities, which results in a better balance between visual perceptibility and quantitative evaluation.

The remainder parts of the study are structured as follows: In Section 2, the proposed image fusion method is systematically explained. The experimental details, quantitative evaluation metrics, qualitative and quantitative results are discussed in Section 3. Section 4 illustrated ablation study and discussion is interpreted in Section 5. The conclusion and the future scope are presented in Section 6.

## 2 Materials and methods

**Fig 1:**
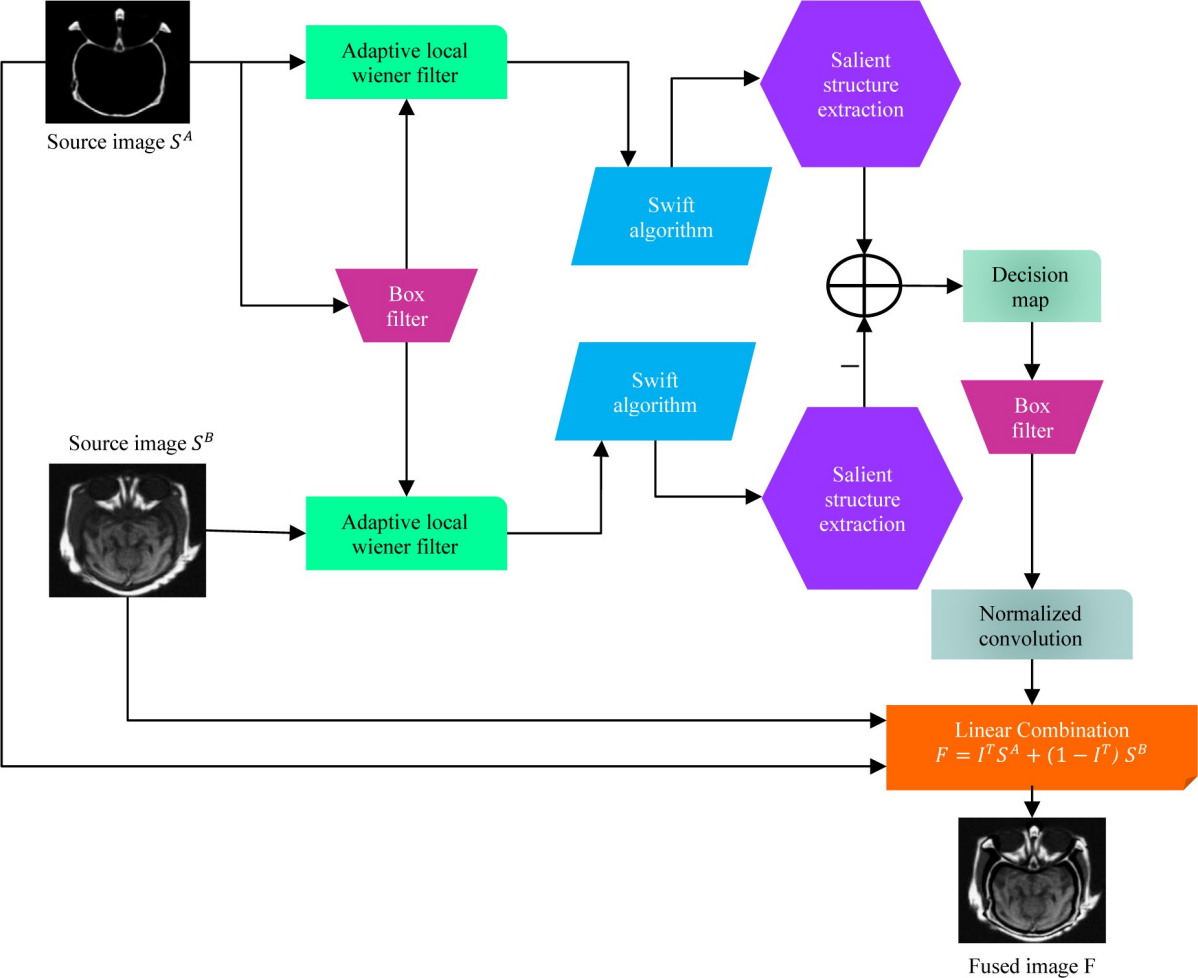
Methodical framework of the proposed method.

This section summarises the overall steps of the proposed fusion method. Fig 1 exhibits schematic flowchart of the proposed method. For simplicity, the fusion approach summarised in this paper assumes that there are two pre-registered source images for integration. The scheme consists of a swift algorithm, salient structure extraction, and normalised convolution (NC). Lastly, the fusion output is obtained by integrating the result of the processed image with the original input images by simple linear combination.

### 2.1 Adaptive local wiener filtering

Assume that the two source images to be combined from different imaging modalities: S^A^ and S^B^. These images are composed of objects at multiple scale where it provides humans with complementary and rich details. The intensities of S^A^ and S^B^ is normalised to remain in the range [0,1]. Assume that each input images S has an amplitude on the range of [0, 255] and carry 8 bits and then S is normalised by:

$$G=normalise\;(S)=\frac{S}{255}$$ (1)

where *G* is a normalised image in the range [0,1]. The input images frequently carry substantial trivial noisy that could attenuate the efficiency to extracts source image large scale structures.

To suppress an effect of noise and unwanted details while maintaining the utmost important details, we employ an improved local wiener filtering. The adaptive local wiener filter [46] is defined as,

$$\bar{G}=\mu_{w}+\frac{\sigma_{w}^{2}-\sigma_{\eta }^{2}}{\sigma_{w}^{2}}(G-\mu_{w}),\;⩝p\in w$$ (2)

where μ_w_ and $\sigma_{w}^{2}$ represents mean and the variance of an image G in the window *w*, respectively, and $\sigma_{\eta }^{2}$ represent constant filter parameter. $\sigma_{\eta }^{2}$ represents estimated noise variance and let constant k represent a constant $\frac{\sigma_{w}^{2}-\sigma_{\eta }^{2}}{\sigma_{w}^{2}}$ then,

$$\bar{G}=\mu_{w}+k(G-\mu_{w}),\;⩝p\in w$$ (3)

We minimise an optimisation problem in w,

$${min}_{k}=\sum_{p\in w}\left({\left(\bar{G}-G\right)}^{2}+\lambda k^{2}\right)$$ (4)

where λ is a regularisation function that penalises larger k. The linear ridge regression method is represented by Eq (4), and the solution is illustrated as

$$k=\frac{\sigma_{w}^{2}}{\sigma_{w}^{2}+\lambda }$$ (5)

### 2.2 Swift algorithm

This algorithm adjusts the contrast of both images and reduces the excessive pixel values [47]. In the pre-processing stage, it eliminates excessive pixel values and achieves a suitable intensity equalisation using a non-complex logarithmic function. Excessive brightness can occur from such extreme levels if they are not controlled appropriately. To find the logarithmic function, one uses the formula [48] as

$$u_{\left(x,y\right)}=\log\left(1+{\bar{G}}_{\left(x,y\right)}\right)$$ (6)

where (*x*,*y*) are the special coordinates, ${\bar{G}}_{\left(x,y\right)}$ is the filtered image with an intensity ranging from 0 to -1, and u_(x,y)_ is the output image following the preprocessing stage. We now have the curve adjustment function, which will be used in the newly built magnitude modification function later. The technique that may be used to compute the amount of variation and distribution in the pairs of pixel scores is the initial adjustment parameter ϛ, which is a corrected sample standard deviation of an image u_(x,y)_. Images with low contrast will have lower **ϛ** scores than images with great contrast, since **ϛ** is a reasonable characteristic to use for contrast in image processing. According to [49], the adjustment factor **ϛ** is calculated as

$$ϛ=\sqrt{\frac{1}{n-1}\sum_{i=1}^{n}{\left(u_{i}-\bar{u}\right)}^{2}}$$ (7)

$$\bar{u}=\frac{1}{n}\sum_{i=1}^{n}u^{i}$$ (8)

where u^i^ stands for vector representation of an image u_(x,y)_ and $\bar{u}$ is the mean of u^i^. Additionally, n is the maximum component size in u_i_. Hence, Eq (9) is used to calculate the second adjustment function η:

$$\eta =\frac{{\left(u_{\left(x,y\right)}\right)}^{\lambda }}{\lambda !}$$ (9)

where λ stands for a tuning function that is initially set to 3 as the default. The next stage in processing an image is to change the contrast and brightness of the image u_(x,y)_. A newly designed nonlinear function involving experimentally determined mathematical, statistical, and geographical features is implemented as part of this process. The process for determining the nonlinear function is as follows:

$$f_{\left(x,y\right)}=\exp{\left(\frac{tan(u_{\left(x,y\right)})-ϛ}{exp(u_{\left(x,y\right)})-\eta }\right)}^{\Gamma }$$ (10)

In this case, u_(x,y)_ is the original image and *tan* is the tangent to the radius of each pixel, and f_(x,y)_ is the improved image with tonality. Here, г is an adjustment factor that controls the amount of enhancement; it must be more than zero. A lower γ value leads to brighter contrast enhancement, while a higher г value leads to less-bright contrast enhancement. Improving an image’s tone is significantly affected by the equation. Accordingly, two distinct curved transformations for filtered images can be produced by employing the tangent in radians in conjunction with elementwise exponential parameters. These two parameters, when combined with adjustment factors ϛ and η, tend to boost brightness and, in certain cases, produce a noticeable changing of tone. The experimental function and values are reduced by two independent adjustment factors ς to counteract these effects. They are both capable of improving the curvilinear transformation that the used functions undergo. The common measure, ϛ can be used in many real-world situations, but η is obtained empirically. Finally, there is the problem of images with limited dynamic ranges and the resulting insufficient image contrast f_(x,y)_. Therefore, to deliver satisfactory quality performance, it needs to be standardised. Therefore, the final stage in post-processing is to employ a regularisation method to restore the image’s pixels to their original dynamic range. As shown in [50], the regularisation operator that was utilised may be identified.

### 2.3 Salient structure extraction

The extraction of salient structures in multiple source images is a main problem in determining which regions of each source image to be selected [51]. Noisy pixels in flat patch could be smoothened by Eq (3), if the regularisation parameter λ is tuned in the range of noise intensity scale, e.g. If G varies more within a window w, we have $\sigma_{w}^{2}>>\lambda ,$ therefore k = 1 and thus $\bar{t}=G$. Suppose G is nearly constant in the window w located on the flat patch, we have $\sigma_{w}^{2}<<\lambda$, therefore k equals to 1 and then $\bar{t}=\mu_{w}$. Thus, main structures such as counters and edges could be maintained and noise in flat areas could be smoothens [52]. The window of filter radius w is set to 3 and the regularisation function is set to λ equals to 0.01, respectively. To approximate the gradient magnitude using structure-detecting approach often by absolute values as

$$M=\left|\frac{\partial \bar{t}}{\partial x}-\frac{\partial \bar{t}}{\partial y}\right|$$ (11)

where x and y represent the special coordinates and the numerical difference is

$$\frac{\partial \bar{t}}{\partial x}=\bar{t}(x+1)-\bar{t}(x).$$

To generate a decision map, the magnitudes of ${\bar{t}}^{A}$ and ${\bar{t}}^{B}$ are compared as

$$D=M^{A}-M^{B}$$ (12)

where M^A^ and M^B^ are the image magnitudes ${\bar{t}}^{A}$ and ${\bar{t}}^{B}$, respectively. D metrics are larger for salient patches and smaller for flat patches. As a result, a large positive score in decision map D represents where a pixel in G^A^ gets sharp altered, whereas a less negative value represents pixels in G^B^ sharp altered. The sharp altered pixel is mostly large-scale structures in images, that is pixel with larger gradient intensity. The mean filter (Eq (13)) is utilised to make sure that pixel with large gradient intensity effects an accord of its neighbours higher and generates the way to spread the sharply altered pixel to its neighbours.


$$\bar{D}=\frac{1}{\left|Ω\right|}\sum_{p\in Ω}D,\;⩝p\in Ω$$ (13)

A binary matrix is the salient structure I^A^,

$$I^{A}=step\;(\bar{D})$$ (14)

In this case, the function step (·) returns 1 for an element of I^A^ if the corresponding element of $\bar{D}$ is positive, and 0 otherwise. Any time the structural matrix I^A^ returns a value of 1, it means that one of the pixels in G^A^ sharp has changed, whereas a value of 0 in G^B^ sharp means the exact opposite.

### 2.4 Edge aware filtering via normalised convolution (NC)

The filtering of non-uniformity sampled image I^A^ in Ω_w_ is analogous to filtering the uniformly sampled signal with missing samples [53]. This scenario has been studied by [54] in the situation of data uncertainty which shows that the proper filtering outputs in mean square scene gets obtains using normalised convolution (NC). The NC defines the filtered score of sample p ∈ D (Ω) for the uniform discretization as

$$I^{T}=(1/K_{p})\sum_{q\in D(Ω)}I^{A}(q)H(t(\hat{p}),t(\hat{q}))$$ (15)

where $K_{p}=\sum_{q\in D(Ω)}H(t(\hat{p}),t(\hat{q}))$ represents normalisation function of p and $t(\hat{p})=ct(p)$. The cost of estimating Eq (16) for all p is O(N)^2^ for N samples and an arbitrary kernel H. However, since ct(x) increases monotonically, we utilise an effective moving average method [55] for performing normalised convolution with box filtering in O(N) time. The box kernel is expressed as

$$H(t(\hat{p}),t(\hat{q}))=\delta \{|t(\hat{p})-t(\hat{q})|\leq r\},$$ (16)

where *r = $\sigma_{H}\sqrt{3}$* represents radius and δ represents Boolean function which returns 1 on the true argument. The box filter has constant radius in Ω_w_, however the space irregularity as well as non-symmetric radius in Ω while its dimension depends on likeness between p and its neighbourhood. This can be interpreted as an estimate where neighbours belong to similar population as p. A box filter is than an advanced estimator of the population mean with correlation to improved anisotropic diffusion [56] and bilateral filter [57]. The cost of running Eq (15) utilising box kernel from Eq (16) is proportional to number of samples. It is used to filtering iterations with $\sigma_{H_{i}}$ defined in [53]. PSNR > 40. When σ_r_ = ∞, the final filter generates an indistinguishable approximation to the gaussian filter after three iterations. An amount of sample adds to or removes from kernel window as its slides from one to another sample is not consistent since samples in w are not uniformly spaced. Thus, box filtering in Ω_w_ necessitates to update K_p_, as well as one extra memory read per sample for checking its domain coordinates. Convolution should only be performed at Ω_w_ positions which contains sample as other locations shall not contributed to the filtered images in an original domain. Lastly, backward differences are used to estimate derivatives.

### 2.5 Obtain the final output by linear combination

To carry out the data transfer, one can use Eq (15), where I^A^ is the input to the normalised convolution and G^A^ is the guidance image. I^A^ includes the input image’s large-scale structures, while G^A^ is an input image that includes items of varying sizes alongside scales. To improve the final fused image’s sharpness and the appearance of the patches along the structures, normalised convolution can be employed to retrieve small-scale features in the vicinity of large-scale structures. A normalised convolution is iterated T times, and the output of this iteration is represented as I^T^. To get the final fused image, we utilise the weighted-sum rule as

$$F=I^{T}S^{A}+(1-I^{T})S^{B}$$ (17)

F is a linear combination of intensities in the two source images *S*_*A*_ and S_B_, and I^T^ shows the weight applied to each source image. The detailed fusion framework has been shown in Fig 1.

## 3 Experimental results and discussion

**Fig 2:**
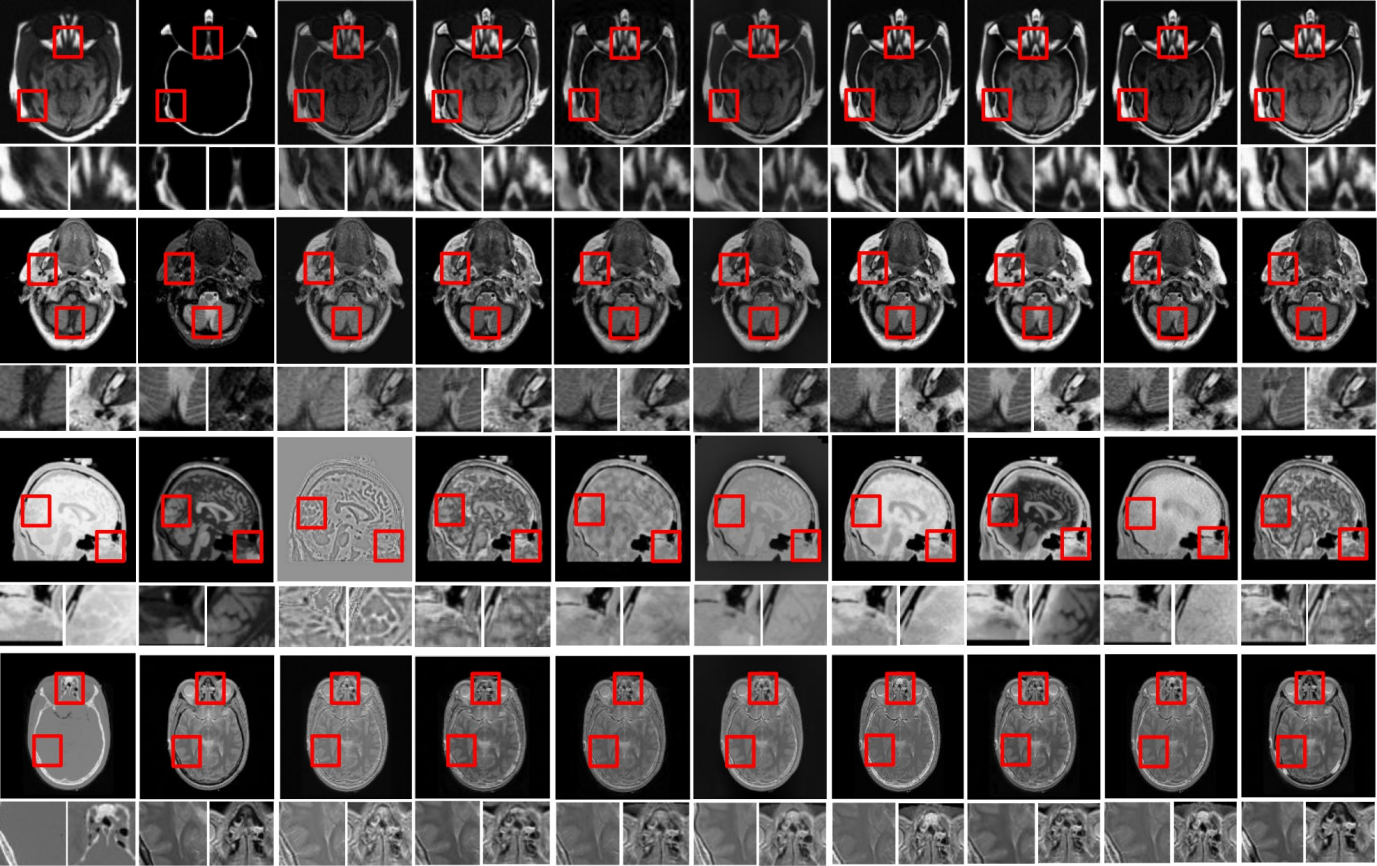
Qualitative comparisons of the proposed algorithm with 7 state-of-the-art algorithms in 4 representative CT-MRI image pairs. From left to right: CT image, MRI image, fusion results of SGF-SR [30], MODE-DNN [33], CNN-NSCT [59], DGF [37],SR-SCNN [35], FDGNet [41], DFENet [42] and the proposed method. For best analysis, two local regions are zoomed as close-ups in each image.

**Fig 3:**
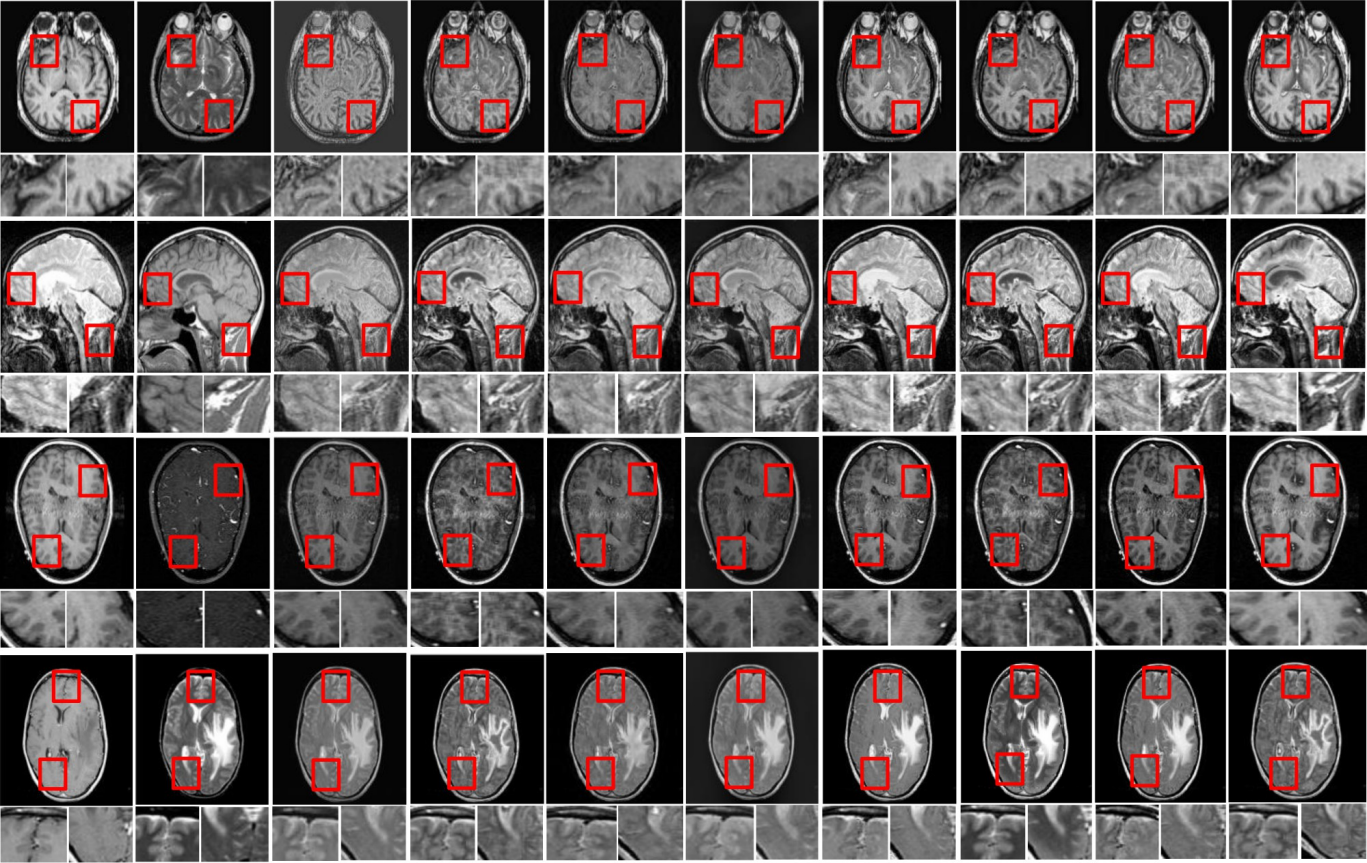
Qualitative comparisons of the proposed algorithm with 7 state-of-the-art algorithms in 4 representative CT-MRI image pairs. From left to right: CT image, MRI image, fusion results of SGF-SR [30], MODE-DNN [33], CNN-NSCT [59], DGF [37], SR-SCNN [35], FDGNet [41], DFENet [42] and the proposed method. For best analysis, two local regions are zoomed as close-ups in each image.

In this study, eight pairs of multimodalities medical images are employed during the experiments to validate the practicability and feasibility of our algorithm [58]. The total of eight sets of CT-MRI images is presented in Figs 2 and 3. All these test datasets contain spatial resolution of 256 × 256 pixel. Further, eight existing comparative methods is utilized for comparison to assess the robustness of our approach including deep guided filtering (DGF) [37], SR-SCNN [35], MODE-DNN [33], **SGF-SR** [30], **CNN-NSCT** [59], FDGNet [41] and DFENet [42]. All the experiments are conducted on PC with Intel (R) Core (TM) i3-7020 CPU @ 2.3 GHz with 8GB RAM. In accordance with [46], the regularization parameter is fixed at λ = 0.01, and radius of filtering window (r) is fix at r = 3 for the pairs of medical images. Further, in [47], the tweaking parameter are set to ϛ = 3.

### 3.1 Quantitative evaluation metrics

The study employs twelve quality evaluation metrics for quantitative assessment to determine the validity of our method, namely spatial frequency (SF), average pixel intensity (API) or mean ($\bar{F}$), mutual information (MI) and fusion symmetry (FS), entropy (H), average gradient (AG), standard deviation (SD), correlation coefficient (CC) [26, 31]. Along with these, also objective fusion metrics based on gradient details is considered [31]. To provide detailed study of fusion efficiency through evaluation of overall fusion efficiency (Q^AB/F^), information loss during fusion process (L^AB/F^) and noise or artifact produced fusion process (N^AB/F^ or $N_{m}^{AB/F}$). The higher values in terms of some metrics and less values in terms of other metrics indicate better fusion quality. The process for computing the parameters and their symbolic representation is given below:

1. Standard deviation represents variance square root and defined as spread of information. It is expressed as
$$SD=\frac{\sqrt{\sum_{i=1}^{m}\sum_{j=1}^{n}(f(i,j)-\bar{F}){)}^{2}}}{mn}$$ (18)
2. Average gradient computes a degree of clarity and sharpness. It is expressed as
$$AG=\frac{\sum_{i}\sum_{j}((f(i,j)-f(i+1,j))2..+(f(i,j)-f(i,j+1))2..)\frac{1}{2}..}{mn}$$ (19)
3. Mutual information computes the total mutual data between a source and the fusion image. It is denoted by:
$$MI={MI}_{AF}+{MI}_{BF}$$ (20)
  where
$${MI}_{AF}=\sum_{k}\sum_{l}p_{A,F}(k,l){log}_{2}(\frac{p_{A,F}(k,l)}{p_{A}(k)p_{F}(l)})$$
$${MI}_{BF}=\sum_{k}\sum_{l}p_{B,F}(k,l){log}_{2}(\frac{p_{B,F}(k,l)}{p_{B}(k)p_{F}(l)})$$
4. Entropy (H) is the information quantity present in the image.
$$H=‐\sum_{k=0}^{255}p_{k}{log}_{2}(p_{k})$$ (21)
  where *p*_*K*_ represent value of intensity probability *k* in image.
5. Spatial frequency computes total information presents in image regions and denoted as
$$SF=\sqrt{{RF}^{2}+{CF}^{2}}$$ (22)
  where
$$RF=\frac{\sqrt{\sum_{i}\sum_{j}(f(i,j)-f(i,j-1))2}}{mn}$$ (23)
  And
$$CF=\frac{\sqrt{\sum_{i}\sum_{j}(f(i,j)-f(i-1,j))2}}{mn}$$ (24)
6. Average pixel intensity (APF) represents index of contrast and represented by
$$API=\bar{F}=\frac{\sum_{i=1}^{m}\sum_{j=1}^{n}f(i,j)}{mn}$$ (25)
  where *m*×*n* represent image dimension and *f*(*i*,*j*) represent intensity of pixel at (*i*,*j*).
7. Correlation coefficient estimates a relevance of representing fusion output to original image. It is defined by
$$CC=(r_{AF}+r_{BF})/2$$ (26)
$$where,\;r_{AF}=\frac{\sum_{i}\sum_{j}(a(i,j)-\bar{A})(f(i,j)-\bar{F})}{\sqrt{(\sum_{i}\sum_{j}(a(i,j)-\bar{A}{)}^{2})(\sum_{i}\sum_{j}(f(i,j)-\bar{F}{)}^{2})}}$$ (27)
$$and\;r_{BF}=\frac{\sum_{i}\sum_{j}(a(i,j)-\bar{B})(f(i,j)-\bar{F})}{\sqrt{(\sum_{i}\sum_{j}(a(i,j)-\bar{B}{)}^{2})(\sum_{i}\sum_{j}(f(i,j)-\bar{F}{)}^{2})}}$$
8. Fusion symmetry represents an amount of symmetry where the fused image is corresponds to input image. It is denoted as
$$FS=2-|\frac{{MI}_{AF}}{MI}–0.5|$$ (28)
9. *Q*^*AB/F*^ = overall information transferred from input images to the fusion result.
10. *L*^*AB/F*^ = overall information loss during fusion process.
11. *N*^*AB/F*^ = noise or artefacts add up in the fused results during the fusion process.

In particular, *Q*^*AB/F*^,*L*^*AB/F*^ and *N*^*AB/F*^ are complementary and summation of all these parameters should result is 1 [31], i.e.,

$$Q^{AB/F}+L^{AB/F}+N^{AB/F}=1$$ (29)

For most of the situations, it has been noticed that the above summation may not lead to the unity. For this purpose, the technique has been modified and the revision has been advised for performance parameters for fusion artefacts. The revised computation is denoted by:

$$N_{m}^{AB/F}=\frac{\sum_{⩝i}\sum_{⩝j}{AM}_{i,j}[\left(1-Q_{i,j}^{AF})w_{i,j}^{A}+(1-Q_{i,j}^{BF})[w_{i,j}^{B}\right)]}{\sum_{⩝i}\sum_{⩝j}(w_{i,j}^{A}+w_{i,j}^{B})}$$ (30)

where ${AM}_{i,j}=\left\{ \begin{array}{l} \;1,g_{i,j}^{F}>g_{i,j}^{A}\;and\;g_{i,j}^{F}>g_{i,j}^{B}\\ 0,\;\mathrm{otherwise} \end{array}\right.$ indicates location of fusion-artefacts where fuse-gradient are powerful than input.

$g_{i,j}^{A},g_{i,j}^{B}\&g_{i,j}^{F}$ represents edge strengths of *A*,*B* and *F*, respectively.

$Q_{i,j}^{AF}$ and $Q_{i,j}^{BF}$ represent gradient details preservation estimates of original images A and B, respectively.

$w_{i,j}^{A}$ and $w_{i,j}^{B}$ represent perceptual weights of original images A and B, respectively.

With the revision of fusion artifacts measure *N*_*m*_^*AB/F*^, we could re-write Eq (28) as

$$Q^{AB/F}+L^{AB/F}+{N_{m}}^{AB/F}=1$$ (31)

### 3.2 Qualitative and quantitative analysis

To investigate the efficiency of our proposed strategy, this study utilizes eight pairs of publicly accessible images from the Whole Brain Atlas [58]. The eight pairs of medical images are presented in Figs 2 and 3. Statistical numeric indicators to validate the objective fusion-performance are considered including MI, H, API, CC, AG, API, SF and SD. Further, in addition to above metrics, an objective fusion characterization based on gradient details are also considered including Q^AB/F^,L^AB/F^,N^AB/F^ and $N_{m}^{AB/F}$ [31].

**3.2.1 Quantitative analysis.** The fused images of MRI and CT dataset of competing methods and the proposed method is presented in Figs 2 and 3. In addition, the two regions of interests (ROIs) (i.e., detail preservation, extracting information, colour fidelity, and so on) is zoomed as a closeup in each image for greater comparisons in Figs 2 and 3. The SGF-SR and MODE-DNN methods both lost a significant amount of energy, resulting in a huge loss in intensities as well as contrast in numerous areas (see bone areas in Fig 2. Although the FDGNet algorithm show better results in this regard, a few areas continue to lose details (see the closeup and the bone areas in Fig 2. The SGF-SR method can preserve image details but fails to extract structural information in MR images (see closeup in Fig 2). The SGF-SR method outperforms the others, but some details are still not extracted. The fused images of the MODE-DNN methods have significant noise artefacts. In all three dataset pairs, our method works suitably in terms of both detail maintenance and extracting details (see Fig 2). Fig 3 portrays image fusion results from MR-CT pairs. The SGF-SR and MODE-DNN methods continue to suffer from the negative effects of detail loss. Further, certain details (i.e., edges) in the MR-CT source images are not properly maintained in the fusion results of these algorithms (see in close-up in Fig 3. The important drawback of the FDGNet approach is its poor ability to extract details. Numerous pieces of information are blurred or even disappear in the fusion results produced by this method (see Fig 3). The fusion results of FDGNet still contain noise-like artefacts. In addition, the FDGNet and SGF-SR methods perform well in general, but there is some magnitude inconsistency in some areas (see Fig 3). Our method produces highly competitive results as compared to other algorithms (see Fig 3). Further, Fig 3 represents the fusion performance of CT-MR images. Because structural information is mainly present in MR images, nearly all these algorithms work well in extracting information, with the main difference being detail preservation. The fusion performance of SGF-SR and MODE-DNN algorithms are severely distorted (see Fig 3). The FDGNet performs better in this regard, but distortion remains in the fusion results of this algorithm (see Fig 3). The FDGNet method handles the details well; however, the spatial In Figs 2 and 3 demonstrates the results of Set A-H image datasets, we observed the visual results of different algorithms. The FDGNet and the proposed method were satisfactory efficient or productive in extracting the maximum functionality characteristics from input images. In case of image pairs, SGF-SR, MODE-DNN and CNN-NSCT loses bone structure information however SR-SCNN, DFENet, DGF, FDGNet and our approach presents desired contrast, though it observed that the fusion performances of SGF-SR, MODE-DNN and CNN-NSCT generate poor-contrasts which make it challenging to clearly differentiate tissues inside the brain. An efficiency of DGF seems to have a superior outcome of contrasting preservation however the edge-information throughout the closeup has been distorted/blurred or smeared and its contrast has been reduced. DFENet-generated fusion findings have enhanced information about the structure and carries appropriate contrasting intensities. However, certain data and contrast information are lost as displayed in the closeup, and the output generated by SR-SCNN lost particulars of soft-tissues (see in Figs 2 and 3). Considering the fusion results of all image pairs, we could notice that the SGF-SR, MODE-DNN, CNN-NSCT and DGF retains information from two source images somehow but nevertheless, it maintains poor contrast. However, by highlighting on the zoomed region in Figs 2 and 3, the bone-skeletal region is completely noticeable in our proposed method. The objective analysis of several fusion algorithms on eight pairs of CT- MRI datasets have been tabulated in Tables 1 and 2. As per the experimental results, the outputs retrieved via proposed method is very efficient and effective in case of all quantitative parameters, whilst other algorithms yielded suboptimal values. Tables 1 and 2 show that our technique ranks first in simulation results for all dataset pairs when compares to other competing techniques. Even if a few parameters are higher with smaller margins than other methodologies, the differences in when compared to existing comparative fusion algorithms, it can be noted that the fusion results for both reference and no-reference based criteria are excellent. Similarly, the visual interpretation of our method for all the image pairs shows better performance. It can be stated to the fact that our algorithm obtains the advantages of normalized weight computation. In addition to that, edge preserving processing and pixel-significance further contributes to computing the optimized weights to generate improved fusion results.

**Table 1:** Quantitative evaluation results of different state-of-the-art methods from Fig 2.

Metrics	Method							
	SGF-SR [30]	MODE-DNN [33]	CNN-NSCT [59]	DGF [37]	SR-SCNN [35]	FDGNet [41]	DFENet [42]	Ours
API	28.7265	33.8837	41.8873	45.9928	48.9916	52.9927	56.8827	60.7525
SD	32.8817	36.8827	40.9833	44.8175	49.8837	51.8736	53.9917	56.8826
AG	6.7625	7.9893	8.7737	10.8837	11.8836	12.8836	12.9667	13.8716
H	4.5515	5.7166	5.9818	6.1663	6.2782	6.7992	7.3442	7.6143
MI	1.9818	2.1983	2.8927	3.8816	3.0991	4.6514	4.6716	4.9715
FS	0.7615	0.8736	0.9355	1.0007	1.1783	1.3881	1.5615	1.6715
CC	0.4671	0.4899	0.5173	0.5637	0.5877	0.6065	0.6254	0.6414
SF	13.7161	14.8817	16.8861	17.9927	19.8816	20.8811	21.8862	22.9917
$Q_{F}^{AB/F}$	0.7614	0.7836	0.8164	0.8366	0.8566	0.8773	0.8936	0.9241
$L_{F}^{AB/F}$	0.1299	0.1062	0.0966	0.0855	0.0688	0.0435	0.0367	0.0156
$N_{F}^{AB/F}$	0.1344	0.1182	0.1076	0.0862	0.0672	0.0462	0.0387	0.0241
$N_{m}^{AB/F}$	0.798	0.0627	0.0562	0.0452	0.0288	0.0167	0.0123	0.0067

**Table 2:** Quantitative evaluation results of different state-of-the-art methods from Fig 3.

Metrics	Method							
	SGF-SR [30]	MODE-DNN [33]	CNN-NSCT [59]	DGF [37]	SR-SCNN [35]	FDGNet [41]	DFENet [42]	Ours
API	56.8826	59.8837	62.8873	66.9937	69.8836	73.9837	86.8983	81.7726
SD	51.7816	53.9937	56.8836	58.1763	60.7761	62.9917	64.1882	66.9917
AG	8.8862	9.8836	10.6763	12.6625	13.8836	14.8881	15.8873	16.8817
H	3.9887	3.8816	4.1883	4.5635	4.6735	5.6171	6.0197	6.6761
MI	2.7715	3.1887	3.5661	3.8927	4.9817	4.6715	5.1542	5.8816
FS	1.5625	1.5985	1.6288	1.6762	1.7299	1.7742	1.8066	1.8817
CC	0.6725	0.7075	0.7566	0.7861	0.8155	0.8355	0.8671	0.8898
SF	19.9917	21.8826	24.9817	25.8827	25.9917	26.9917	27.6615	28.9917
$Q_{F}^{AB/F}$	0.7718	0.8065	0.8266	0.8455	0.8625	0.8826	0.9060	0.9167
$L_{F}^{AB/F}$	0.1067	0.0807	0.0672	0.0562	0.0452	0.0389	0.0267	0.0176
$N_{F}^{AB/F}$	0.1267	0.1063	0.0856	0.0762	0.0672	0.0572	0.0355	0.0213
$N_{m}^{AB/F}$	0.617	0.0572	0.0467	0.0300	0.0297	0.0198	0.0122	0.0064

**Fig 4:**
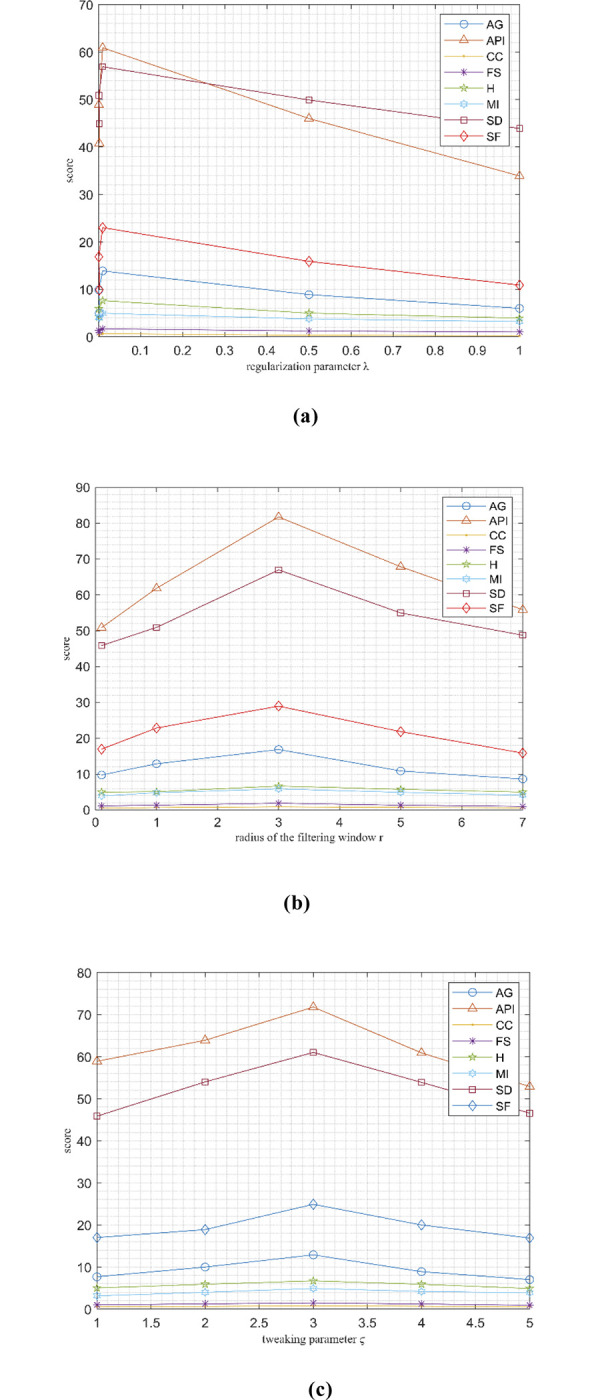
Impacts of the trade-off parameters λ, r and ϛ on quantitative performance of proposed method.

**3.2.2 Quantitative analyses.** In addition to above eight performance metrics, further the results of four fusion performance metrics based on gradient details has been discussed in this section. These metrics provides detailed evaluation of fusion results through evaluating overall details transfers from source images to fusion result Q^AB/F^, the comprehensive information loss L^AB/F^, noise and artifacts is added in the fusion images during the fusion procedure N^AB/F^ and revised fusion artefacts measure $\left(N_{m}^{AB/F}\right)$. We utilized these metrics to compares our algorithm with other competing approaches to show the performance of overall methods. For fusion comparison, the proposed technique is compares with seven other well-known recent fusion methods that includes SGF-SR, MODE-DNN, CNN-NSCT, DGF, SR-SCNN, FDGNet and DFENet in Tables 1 and 2 demonstrates the quantitative fusion results on gradient details metrics. The detailed information is presents in SGF-SR, MODE-DNN, FDGNet, DFENet and DGF methods. The detailed information retention and brightness of fused images DFENet are desirable then FDGNet and SR-SCNN (see in Figs 2 and 3). The DFENet method are more capable to preserve edge details. The textures and the edges in the fusion results by the proposed technique has been efficiently maintained. In Tables 1 and 2, it shows that our algorithm nearly always gets ideal values on all objective measures as compared to other competing algorithms which intuitively adapt at quality judgements than other algorithms. In summary, the fusion performance of our strategy surpasses all other algorithms is case of both subjective interpretation and quantitative assessment. The overall performance measures of evaluation metrics are shown in Tables 1 and 2 where the fusion performances of our algorithm are superior with ideal scores in all these eight-objective metrics. The principal aims of image fusion is to produce an output that is in highly suitable for human visualization with adequate, comprehensive, and detailed features the fusion results. Further, visual interpretation is quite important along objective performance. The fusion results are demonstrated in Figs 2 and 3, which interprets performance visually. The proposed method has demonstrated better visualization when compares to other comparable algorithms. The information transfer rate in the proposed technique is the highest when comparison to other algorithms according to the quantitative assessment in case of gradient based measures. In addition, the suggested method has the high Q^AB/F^ and small $L^{AB/F},N^{AB/F}N_{m}^{AB/F}$ across all pairs of images when comparison to other competing algorithms which is ideal for better performance. In addition, the effect of parameters such as adjustment parameter (**ϛ)**, tweaking parameter (**λ)** and range of frequency (r) is shown in Fig 4. We observed that, variation in these parameters affects the performance of the proposed method. In this study, we fixed the optimal values for all these parameters for entire experiments.

## 4. Ablation study

In this section, comparative experiments are conducted to study the impact of Salient structure extraction (SSE), Swift algorithm, and normalized convolution.

### a. Salient structure extraction

This approach provides an easy-to-implement technique for extracting salient features from images by making use of image gradients to get large-scale structures of the source images. Furthermore, the structure-preserving filter can restore small-scale details from the reference image about large-scale structures in the input images. The output of the structure-preserving filter and the source images are combined using a weighted-sum rule to produce the fusion result, which is based on the property of the structure-preserving filtering as a weighted map. The noisy pixels in flat patches can be smoothed by adjusting the regularization parameter λ within a range of the noise intensity scale. That is, if *G*_*p*_ undergoes significant changes within the window *ω*, with $\sigma_{\omega }^{2}\gg \lambda$ and *k* = 1, then ${\bar{G}}_{p}$ is equal to *G*_*p*_. We have $\sigma_{\omega }^{2}\gg \lambda$, thus *k* = 0, and ${\bar{G}}_{p}=\mu_{\omega }$ if *G*_*p*_ is almost constant in a window ω situated at a flat patch. This way, we can smooth out noise in flat areas while preserving the main features such as edges and corners. The regularisation parameter λ is set to *λ* = 0.01, and the radius of the filtering window is set to 7, correspondingly [60–62].

### b. Swift algorithm

This algorithm is employed to improve the contrast of low-contrast images. As a first step in pre-processing, it applies a non-complex logarithmic function to reduce the immoderate pixel values. A non-linear enhancement function is used to modify the contrast and brightness. This function was constructed using mathematical, statistical, and spatial information. Lastly, the image pixels are rearranged into their natural dynamic range as a post-processing step using a regularization function.

### c. Normalized convolution

This convolution establishes an isometry between the real line and curves on the 2D image manifold. To efficiently execute 1D edge-preserving filtering in linear time, it adaptively warps the input signal while retaining the geodesic distances between points on the curve. Using 1D operations results in significant speedups compared to existing techniques and potential memory savings; the computing cost is not affected by the choice of the filter parameters.

## 5 Discussion

It can be noticed that the suggested algorithm’s non-reference-based measures values is higher than results obtained from other competing approaches. The visual interpretation is perceived to a significant extent with the approach outlined in the manuscript. According to the results obtained from the Tables 1 and 2, the fusion results of four methods named as SGF-SR, MODE-DNN, CNN-NSCT and DGF, we can see the fusion performance of mentioned methods suffered from distortions as well as the soft tissues of are missing (see in Figs 2 and 3. The contrast of the fusion outputs from both SR-SCNN, FDGNet and DFENet are slightly lesser than our technique (see in Figs 2 and 3. Same as SGF-SR, MODE-DNN, CNN-NSCT and DGF, the soft tissues of original images by FDGNet and DFENet methods is slightly missing (see in Figs 2 and 3). The contrast of SR-SCNN algorithm is slightly low as compares to our method. Thus, in case of all evaluation aspects, our algorithm presents desirable fusion results in terms of clinical applicability. Tables 1 and 2 validates the quantitative evaluation results of several fusion algorithms on three sets of CT-MRI photographs. The average value of each algorithm across all testing datasets in an individual fusion scenario is tabulated. The highest value demonstrated in bold for each metric represents the best value among all six algorithms, and we also underline the values in second-best. The contents of Table 1 can be compared to Figs 2 and 3 to provide a more intuitive understanding of the objective results of various fusion algorithms. Overall, the proposed strategy is the only one among six comparative methods which present consistent ranks for all four measures and all pairs of images, demonstrating the high quality of our method. For all image pairs, the proposed method secures first place on the metrics SD, SF, Entropy. In comparison to other methods, Table 1, and Figs 2 and 3 show that our method outperforms all these algorithms on almost all measures across all pairs of images. In addition, Table 3 presents the computational time for execution of all methods (i.e., DGF [37], SR-SCNN [35], MODE-DNN [33], SGF-SR [30], CNN-NSCT [59], FDGNet [41] and DFENet [42] and the proposed method). We can see that the CNN-NSCT, SR-SCNN, FDGNet and DFENet methods have the longest running times. This is so because deep learning models takes longer processing time during training and testing phase. Therefore, it observed that, the proposed method requires lesser execution time, comprise complexity and requires less space compared to all other state-of-the-art methods. The proposed method is satisfactory owing to its delivery of good performance with lower resource usage i.e. it requires very less amount of data to process. Further, this method implementation requires significantly lower CPU resources and about half of the memory bandwidth. Furthermore, this method performs a multimodal image fusion operation with low computational complexity due to simple algorithm/framework as shown in Fig 1.

**Table 3:** Average running times for the different algorithms.

Method	Average running time (s)
SGF-SR [30]	0.0513
MODE-DNN [33]	0.0567
CNN-NSCT [59]	0.0962
DGF [37]	0.0527
SR-SCNN [35]	0.0784
FDGNet [41]	0.0915
DFENet [42]	0.1026
Ours	0.0486

## 6 Conclusion and future work

This paper presents a novel method to fuse medical images that combines the benefits of salient structure extraction (SSE) with a swift algorithm for CT-MRI images using normalized convolution. The method proposes a gradient-based scheme for the extraction of salient structures from multimodality medical images. The method utilizes a swift algorithm for reducing excessive pixel values and modifying image brightness and contrast. It further implements high-quality edge-aware image filtering using a dimensionality reduction method. It leads to filters with different desirable properties and notably speeds up the fusion operation. In addition, the output of normalized convolution is combined with the source images using a weighted-sum rule to produce the fusion result, which rearranged image pixels into their original dynamic range for restoring large-scale structures. The results are demonstrated with eight pairs of CT-MR images to ensure the effectiveness of our method. The results of a comparison of five state-of-the-art fusion methods show that our method obtains comparable results both visually and in quantitative evaluation metrics. Our approach consists of low computational complexity and less execution time while improving diagnostic computing accuracy. Our method exhibits a major boost in efficiency when compared to other state-of-the-art algorithms. It can be observed that the qualitative interpretation of all fusion images retrieved through the proposed algorithm has superior visual quality. In the future, we will focus on developing highly efficient fusion models to further enhance the fusion results. Besides, we will explore the prospects of our method for other image fusion problems. The proposed method could be augmented by using other different types of filters that have been left for future research. Further, image fusion and the importance of this technique have huge prospects for improvement by proposing several image fusion algorithms to decrease noise and artifacts. In addition, it is quite realistic to boost the superiority of medical images by further improving the proposed algorithm in the future.
